# Hypoxia-inducible factor-prolyl hydroxylase inhibitors for treatment of anemia in chronic kidney disease: a systematic review and network meta-analysis

**DOI:** 10.3389/fphar.2024.1406588

**Published:** 2024-07-10

**Authors:** Song Ren, Yurong Zhao, Jingyu Wu, Shangqing Ren, Yunlin Feng

**Affiliations:** ^1^ Department of Nephrology and Institute of Nephrology, Sichuan Academy of Medical Sciences and Sichuan Provincial People’s Hospital, School of Medicine, Sichuan Clinical Research Centre for Kidney Diseases, University of Electronic Science and Technology of China, Chengdu, China; ^2^ Robotic Minimally Invasive Surgery Center, Sichuan Provincial People’s Hospital, University of Electronic Science and Technology of China, Chengdu, China

**Keywords:** hypoxia-inducible factor-prolyl hydroxylase inhibitor, anemia, chronic kidney disease, efficacy, safety, network meta-analysis

## Abstract

**Purpose:**

To review current evidence on the efficacy and safety outcomes of HIF-PHIs in chronic kidney disease (CKD) populations with an emphasize on the safety profile.

**Methods:**

A systematic search was conducted in the Medline, Embase, and Cochrane Central databases. Randomized controlled trials that had assessed the efficacy and safety of HIF-PHIs for anemia in CKD were included. The efficacy outcome included change of hemoglobin and the safety outcomes any adverse events, severe adverse events, major adverse cardiovascular events, and mortality. The qualities of studies were assessed using the Cochrane ROB tool.

**Results:**

47 studies encompassing 55 RCTs for the study outcomes were included in this study. All six commercially available HIF-PHIs had direct comparisons to ESA and placebo, yet lacked direct comparisons among each other. The network analysis demonstrated all six HIF-PHIs were able to effectively elevate hemoglobin in the general CKD patients compared to placebo. All HIF-PHIs did not differ among each other in the efficacy of correcting anemia. Roxadustat and daprodustat had the largest number of reports in terms of adverse events. The overall risk of each safety outcome did not increase in comparison to erythropoiesis stimulating agent (ESA) or placebo, and did not differ among different types of HIF-PHIs.

**Conclusion:**

HIF-PHIs can effectively elevate hemoglobin without causing higher risk of safety concerns in CKD patients with anemia. Further evidence from long-term studies and the ongoing post-market surveillance is necessary.

## Introduction

Anemia is a prevalent condition observed in a significant number of individuals with chronic kidney disease (CKD) and plays a crucial role in the ongoing management of CKD (Stauffer and Fan, 2014; Li et al., 2016). Despite advancements in the treatment of renal anemia through the use of erythropoiesis stimulating agents (ESAs) and iron supplements (Coyne et al., 2011; Litton et al., 2013), there remains a subset of patients for whom the correction of hemoglobin levels proves challenging (Coyne et al., 2011). Additionally, the need of transfusion raises the risk of allograft rejection in the future kidney transplantation.

Although the superiority of hypoxia-inducible factor-prolyl hydroxylase inhibitors (HIF-PHIs) in reducing transfusion needs in comparison to ESAs has not been supported by evidence, HIF-PHIs have significantly transformed the therapeutic approach to renal anemia (Besarab et al., 2015) and undergone notable advancements in recent years, helping to address the requirements of correcting anemia in CKD patients (Borawski et al., 2021; Mima, 2021; Natale et al., 2022). Due to its distinct underlying mechanisms, HIF-PHIs have garnered substantial evidence supporting their efficacy in correcting anemia, mainly in CKD, as well as in hematological disease in scattered reports (Mima, 2021; Chen et al., 2023; Yang et al., 2023).

Despite the demonstrated positive effects of HIF-PHIs on hemoglobin levels and iron metabolism (Souza et al., 2020), the potential cardiovascular risks and elevated VEGF levels associated with their use have been a topic of ongoing discussion (Guimarães et al., 2023). Four network meta-analyses have been published regarding the effectiveness and safety of HIF-PHIs in treating renal anemia (Zheng et al., 2020; Fadlalmola et al., 2022; Chen et al., 2023; Yang et al., 2023); however, these network meta-analyses treated dialysis and non-dialysis dependent patients separately and primarily emphasized the efficacy of HIF-PHIs on hemoglobin and iron metabolism, with limited attention given to their safety profile. The efficacy and safety profiles in the overall CKD population are still to be illustrated.

Therefore, we undertook a comprehensive review and network meta-analysis of randomized controlled trials to evaluate the effectiveness and safety of HIF-PHIs in individuals with CKD, with a particular focus on assessing the safety profile. The aim of this study was to gain a thorough understanding of the existing evidence and offer valuable insights for clinical practitioners.

## Methods

### Data sources and literature search

We conducted a systematic literature search according to the Preferred Reporting Items for Systematic Review and Meta-Analyses (PRISMA) statement (Liberati et al., 2009) from inception through 13 September 2023 in MEDLINE via PubMed, EMBASE via Ovid, and Cochrane Central Library, using text words and medical subject headings (MESHs) relevant to “hypoxia-inducible factor-prolyl hydroxylase inhibitor,” “kidney disease,” and “randomized controlled trials” that were combined using Boolean search terms “AND” and “OR” (Supplementary Table S1). The search was limited to studies published in English. This systematic review and network meta-analysis has been registered in PROSPERO (Identifier# CRD42023429560).

### Outcomes

The outcomes in this meta-analysis encompassed efficacy and safety outcomes. The efficacy outcome referred to the effect of HIF-PHIs to correct renal anemia, either increase hemoglobin or maintain hemoglobin in target ranges. The safety outcomes referred to any adverse events (AE), severe adverse events (SAEs), major adverse cardiovascular events (MACEs), and mortality.

### Study selection

Two reviewers (S.R. and Y.R.Z.) independently conducted the study selection following a standardized approach. Titles and abstracts of records returned from the literature research were carefully examined. The remaining articles then underwent a full-text review for further exclusion. Reference lists of review articles were also manually screened for eligible studies that could have been missed.

Only randomized controlled trials that had assessed the efficacy and safety of HIF-PHIs for anemia in CKD were considered eligible for this study. Studies were excluded if they were: 1) duplicates, 2) reviews, protocols, comments, or editorials; 3) conducted in pediatric population; 5) cohort observational studies; and 6) animal model or *in vitro* studies. Studies without information on the study outcomes were also excluded. Any discrepancy was resolved through discussion and also adjudication from a third reviewer (Y.L.F.).

### Data extraction

Data was extracted by two reviewers (S.R. and S.Q.R.) independently using Microsoft Excel spreadsheet and compiled onto a single one after cross examination. Any disagreement was resolved by the third reviewer (Y.L.F.). The extracted data included authors, publication year, geographic region, targeted population, sample size, numbers of patients and the detailed regimen in the experimental and control groups, and outcomes.

### Quality assessment

The risk of bias was independently assessed by two reviewers (S.R. and Y.R.Z.) based on the “Cochrane Handbook for Systematic Reviews of Interventions” imbedded in the RevMan analysis software (2022) (Higgins et al., 2022). Any discrepancy was resolved by the third reviewer (Y.L.F.). Risk of bias was analyzed for all studies and each individual study separately.

### Data synthesis and analysis

The STATA (version 17.0; Stata Corporation, TX, United States) software were used for data synthesis and analysis. To evaluate continuous outcomes, the changes following treatment in comparison to baseline were used. Changes in the studies that had only reported the results before and after treatments were calculated by subtracting the baseline value from the pre-treatment value prior to data synthesis. The meta-analysis for continuous outcomes included direct comparisons for each pair of treatments and the network meta-analysis for multiple comparisons including indirect comparisons via pooled mean differences (MD) with 95% confidence intervals (CIs) using a random-effects model. Network map was used to shown the interactions among different treatments. The meta-analysis for categorized outcomes followed a similar procedure and utilized pooled Odds Ratios (ORs) with 95% CI using a random-effects model. The treatments were sorted in rank based on surface under the cumulative ranking curve (SUCRA) (Salanti et al., 2011) for each outcome and graphically illustrated using the ranking panel plots. The higher the rank, the superior the treatment effect. Statistical heterogeneity was estimated using the I^2^ statistic, for which an I^2^ value of <25%, between 26% and 75%, and >75% represents low, moderate, and high heterogeneity, respectively (Ioannidis, 2008). The assumption of consistency in the network analysis was verified using a design-by-treatment approach (Higgins et al., 2012). A two-sided *p*-value of <0.05 was considered statistically significant. Publication bias was assessed by visual inspection of the comparison adjusted funnel plot.

## Results

### Characteristics of the included studies

**FIGURE 1:**
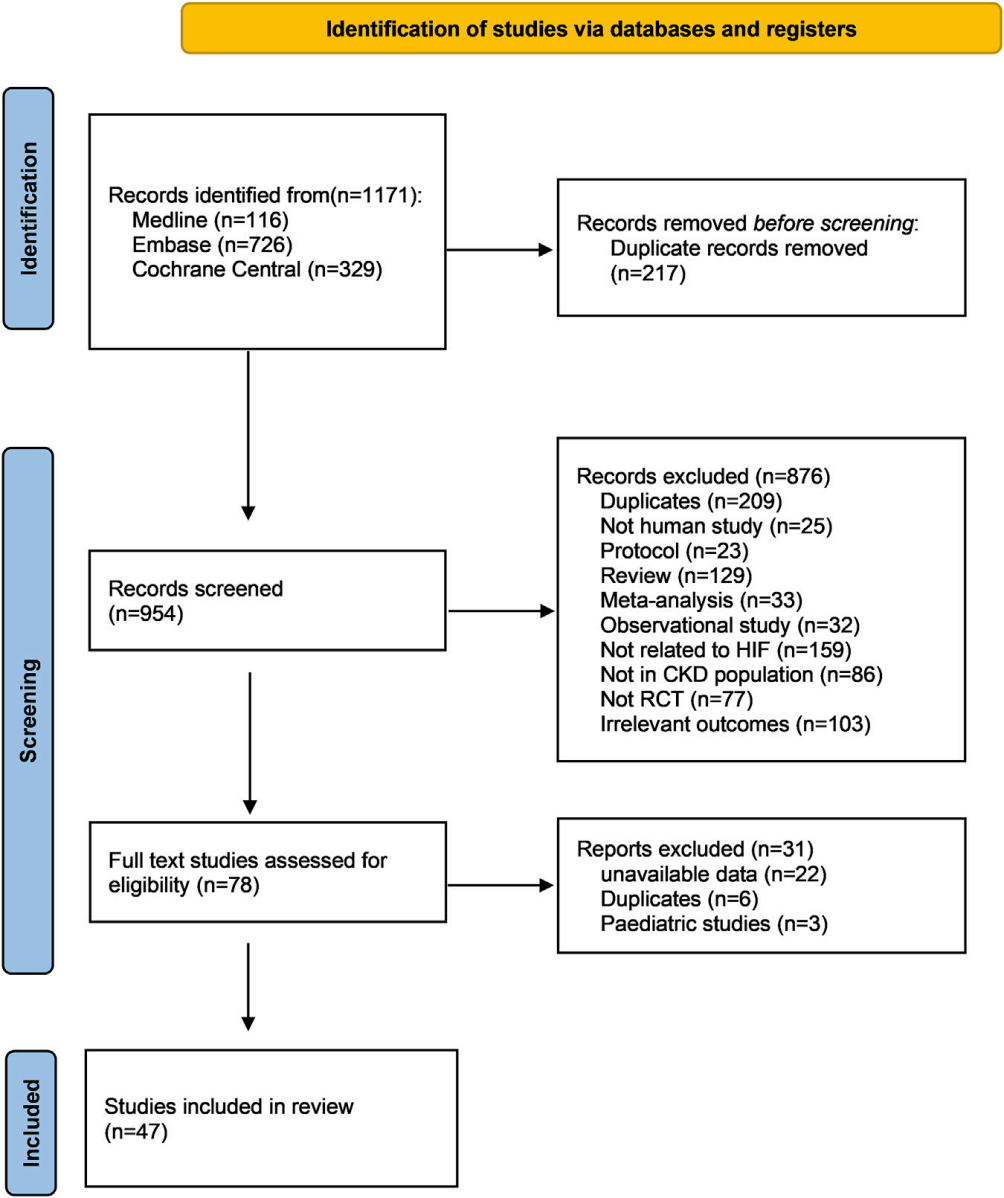
PRISMA flow chart of this network meta-analysis. Abbreviations: CKD, chronic kidney disease; RCT, randomized controlled trial.

A total of 954 records were obtained from the literature search following the elimination of duplicate entries. Subsequent to the evaluation of titles and abstracts, 78 publications were deemed suitable for full text review, of which 31 were further excluded, resulting in 47 studies for inclusion in this network meta-analysis (Agrawal et al., 2022; Akizawa et al., 2019a; Akizawa et al., 2020a; Akizawa et al., 2019b; Akizawa et al., 2019c; Akizawa et al., 2019d; Akizawa et al., 2021a; Akizawa et al., 2021b; Akizawa et al., 2020b; Akizawa et al., 2017; Akizawa et al., 2021c; Bailey et al., 2019; Barratt et al., 2021a; Barratt et al., 2021b; Brigandi et al., 2016; Charytan et al., 2021; Chen et al., 2019a; Chen et al., 2019b; Chen et al., 2017; Chertow et al., 2021; Coyne et al., 2021; Coyne et al., 2022; Csiky et al., 2021; Eckardt et al., 2021; Fishbane et al., 2021; Fishbane et al., 2022; Gang et al., 2022; Holdstock et al., 2019; Holdstock et al., 2016; Hou et al., 2022; Martin et al., 2017; Meadowcroft et al., 2019; Nangaku et al., 2020; Nangaku et al., 2021a; Nangaku et al., 2021b; Nangaku et al., 2021c; Parmar et al., 2019; Pergola et al., 2016; Provenzano et al., 2016; Provenzano et al., 2021; Shutov et al., 2021; Singh et al., 2021; Singh et al., 2022; Yamamoto et al., 2021b) (Figure 1).

All studies were published from the year 2015 onwards. Among these, 43% (20 out of 47) were published in the year 2021. Geographically, the studies were distributed as follows: 23 in Asia, 19 in North America, and five in Europe. 22 studies were conducted in dialysis dependent patients and the other 25 were conducted in non-dialysis dependent patients. The follow up duration ranged widely from 4 to 240 weeks. Among these 47 studies, six studies reported 2 RCTs and one study reported 3 RCTs, adding up to a total number of 55 RCTs for the final comparisons for different outcomes. The detailed characteristics of included studies were shown in Supplementary Table S2.

### Efficacy of HIF-PHIs on hemoglobin

**FIGURE 2:**
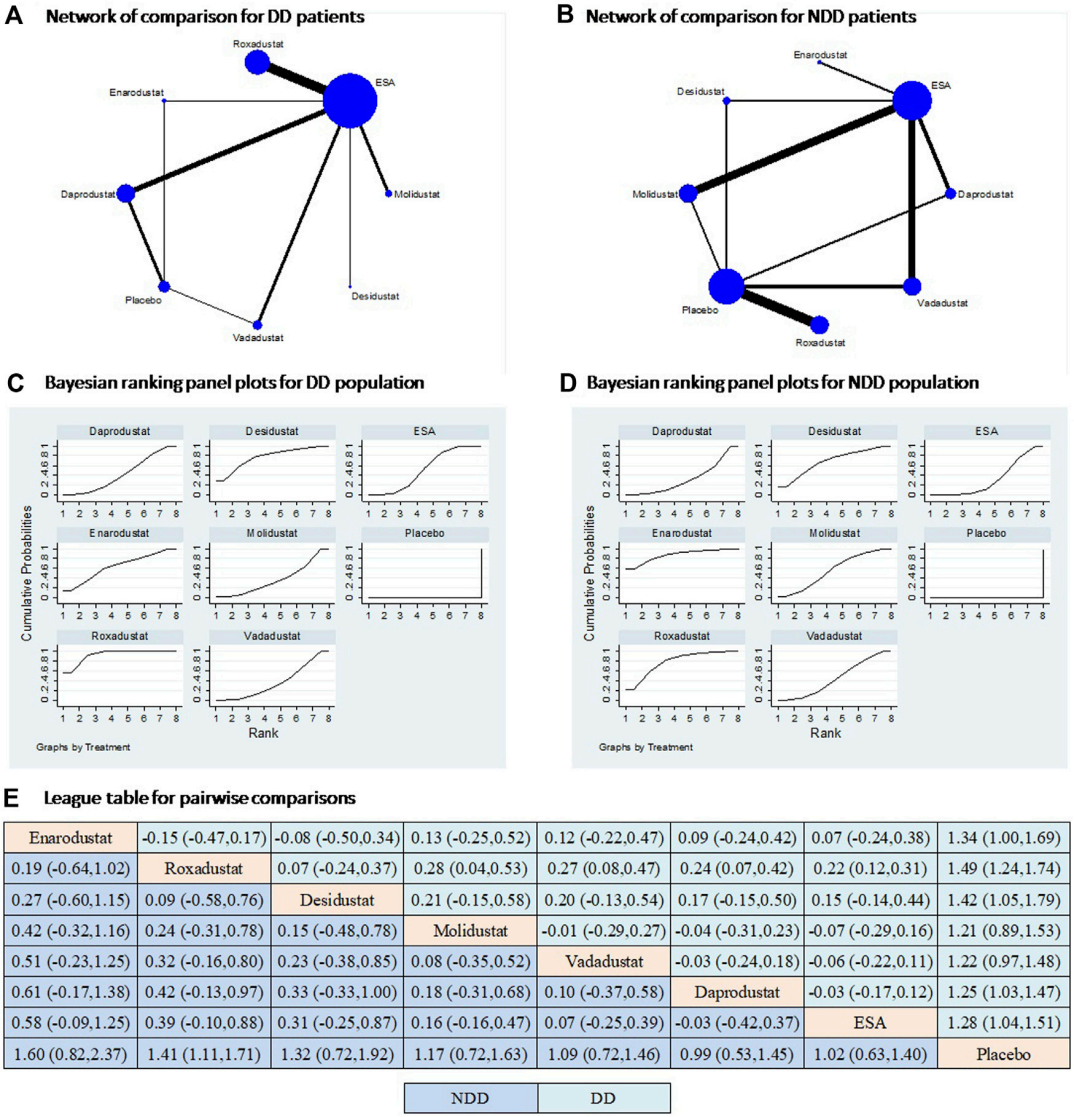
Comparison for the efficacy on hemoglobin following different treatments in dialysis and non-dialysis dependent populations. Note: **(A and B)** In the network of comparisons, the size of nodes is proportional to the total sample size of each treatment, and the width of lines is proportional to the number of studies in each pair of comparison. **(C and D)** Bayesian ranking panel plots indicate the higher the rank reflected by the area under curve, the superior the treatment to increase the levels of hemoglobin. **(E)** The league table of pairwise comparison for the effects of different treatments on hemoglobin levels. All treatments are ordered based on efficacy ranking. Abbreviations: DD, dialysis dependent; NDD, non-dialysis dependent; MACE, major adverse cardiac events.

All six HIF-PHIs were compared to either ESA or placebo in the study. There is a lack of direct comparison between the different HIF-PHIs in both dialysis-dependent (DD) and non-dialysis-dependent (NDD) populations (Figure 2). The results of direct comparisons supported the efficacy of HIF-PHIs to elevate hemoglobin over the placebo; however, these advantages were not evidently observed in comparison to ESA treatment (Supplementary Figure S1). Among all the HIF-PHIs that reported an effect on hemoglobin levels, roxadustat had the largest total sample size. Roxadustat demonstrated a non-inferiority in increasing hemoglobin levels compared to ESA in DD populations, and to ESA and placebo in NDD populations. Enarodustat, desidustat, and daprodustat showed non-inferiorities in comparison to placebo in DD populations (Figure 2). Overall, the effects of all HIF-PHIs on hemoglobin levels did not significantly differ from each other. A compiled analysis in both dialysis and non-dialysis dependent populations yielded similar findings (Supplementary Figure S2).

### Safety of HIF-PHIs

**FIGURE 3:**
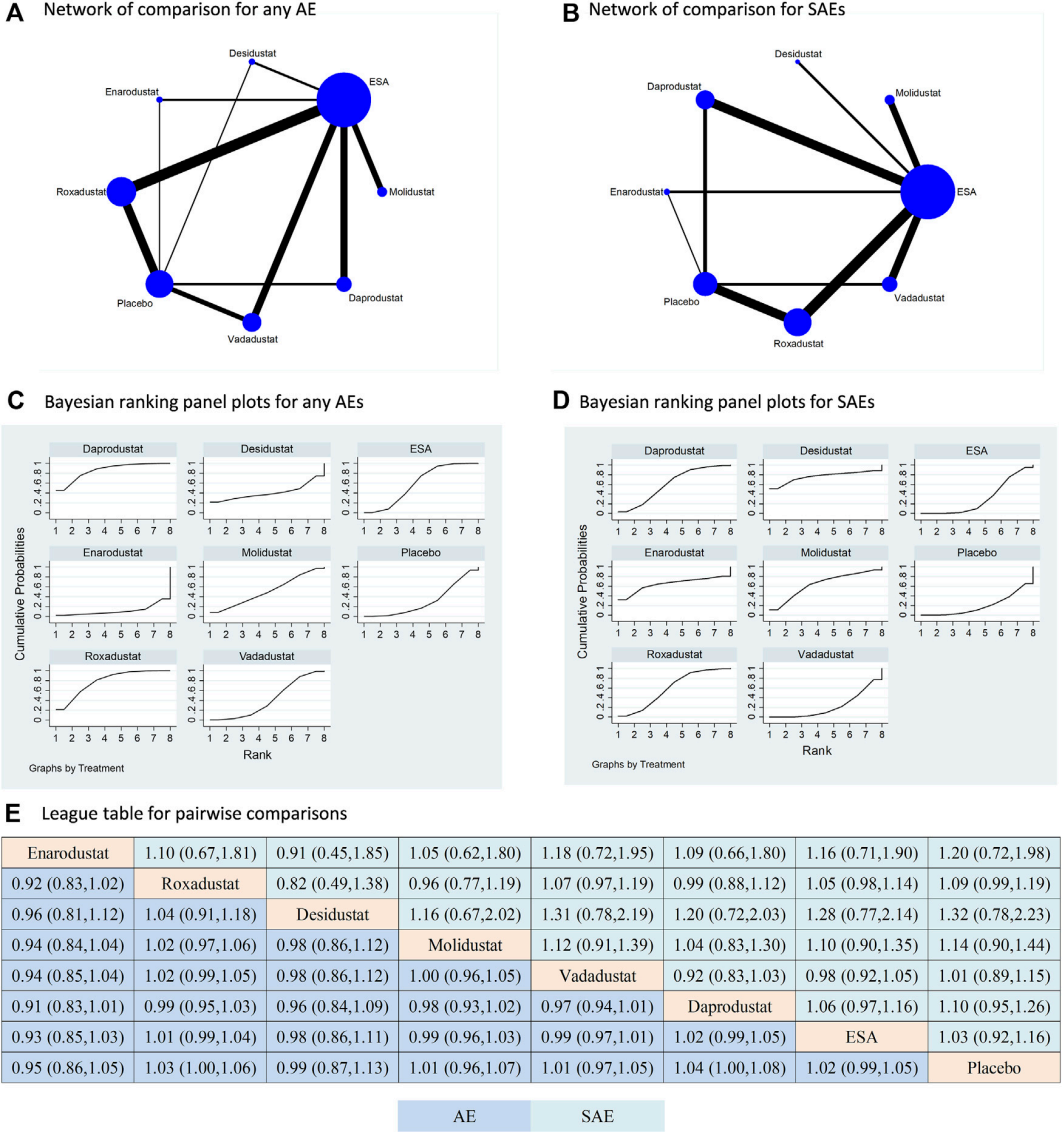
Comparison for the risk of any adverse event and severe adverse event following different treatments. Note: **(A) (B)** In the network of comparisons, the size of nodes is proportional to the total sample size of each treatment, and the width of lines is proportional to the number of studies in each pair of comparison. Bayesian ranking panel plots indicate the higher the rank reflected by the area under curve, the superior the treatment to increase the risk of any AE **(C)** or SAE **(D)**. **(E)** The league table of pairwise comparison for the risk of any AE and SAE following different treatments. All treatments are ordered based on efficacy ranking.

All studies included in the analysis reported outcomes of any AE and SAE; however, four studies specifically focusing on enarodustat did not provide information on MACE or mortality (Akizawa et al., 2019c; Akizawa et al., 2019d; Akizawa et al., 2021a; Akizawa et al., 2021b). As a result, the comparisons of any AE and SAE included six types of HIF-PHIs, while the comparisons of MACE and mortality only involved five types of HIF-PHIs. In terms of both any AE and SAEs, all HIF-PHIs were compared to ESA or placebo. Notably, roxadustat demonstrated the strongest evidence, as indicated by its largest total sample size and the highest number of RCTs (Figure 3A,B). There is a lack of direct comparisons between HIF-PHIs. ESA is once again the prevailing reported controlled therapy. In relation to placebo, all HIF-PHI treatments exhibited no significant elevation in the risk of any AE or SAE, and there was no discernible variation in risk between any two categories of HIF-PHIs (Figure 3C–E). These findings are corroborated by the direct comparisons (Supplementary Figure S3).

**FIGURE 4:**
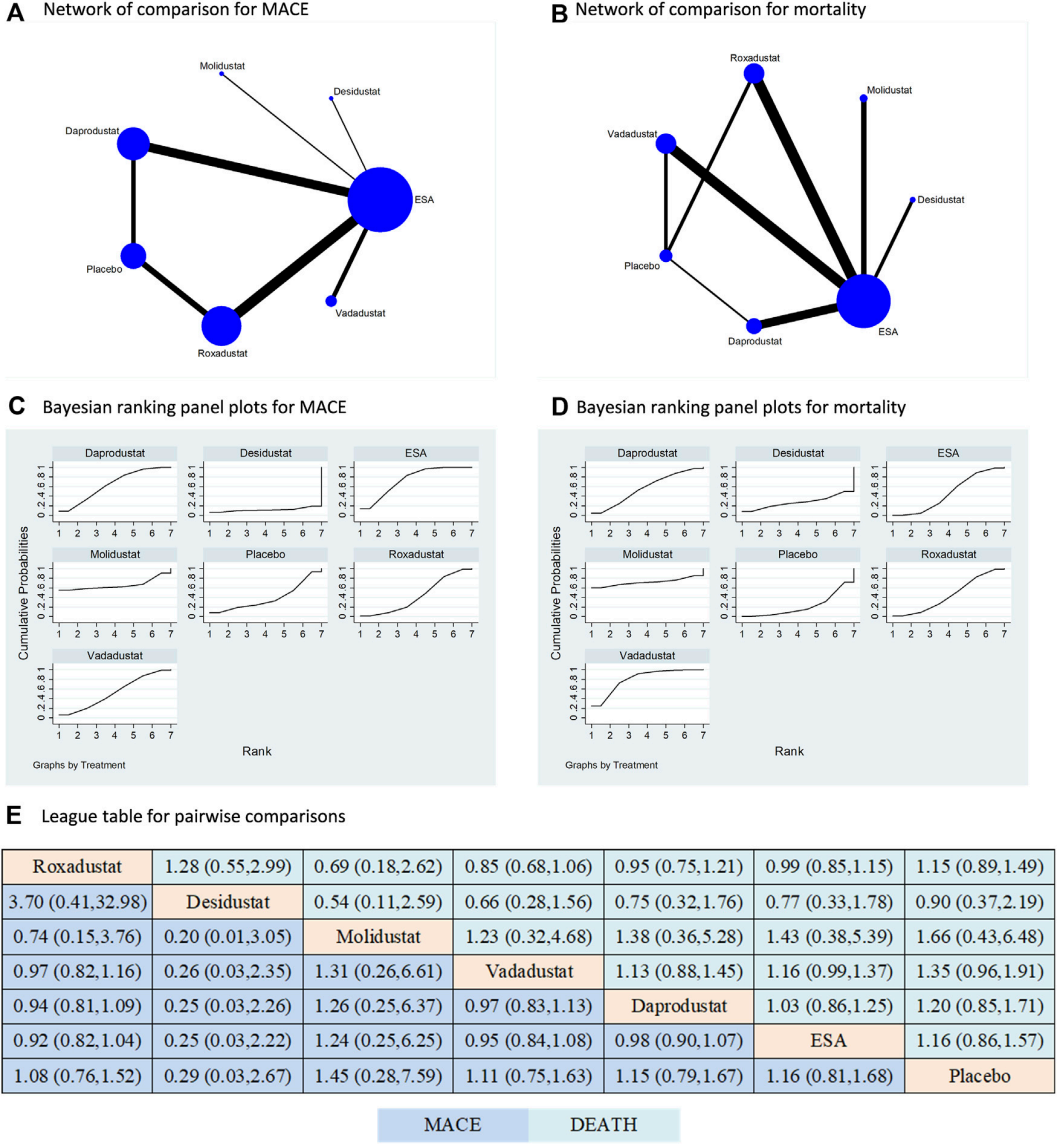
Comparison for the risk of MACE and mortality following different treatments. Note: **(A) (B)** In the network of comparisons, the size of nodes is proportional to the total sample size of each treatment, and the width of lines is proportional to the number of studies in each pair of comparison. Bayesian ranking panel plots indicate the higher the rank reflected by the area under curve, the superior the treatment to increase the risk of MACE **(C)** or mortality **(D)**. **(E)** The league table of pairwise comparison for the risk of MACE and mortality following different treatments. All treatments are ordered based on efficacy ranking.

Five types of HIF-PHIs had been compared to ESA or placebo in terms of both MACE and mortality; however, there is still absence of direct comparisons among the different types of HIF-PHIs (Figure 4A,B). ESA was the most commonly used controlled therapy in terms of both sample size and number of studies. Similar to the findings for any AE and SAE, the HIF-PHI treatments did not significantly increase the risk of MACE or mortality compared to placebo or ESA. Furthermore, there was no significant difference in risk between any two types of HIF-PHIs (Figure 4C–E). The findings are additionally supported by the direct comparisons (Supplementary Figure S4).

#### Consistency assessment

Consistency test results demonstrated consistency among the direct and indirect comparisons for the efficacy and all safety outcomes (Supplementary Table S3).

#### Publication bias

Visual inspections of the funnel plots of the effect on hemoglobin and safety outcomes in the included studies revealed absence of asymmetry for the efficacy and safety outcomes (Supplementary Figures S5 and S6).

#### Risk of bias assessment

Critical appraisal indicated 15, 34, and 6 RCTs were rated as having low, high, and unclear risk of bias based on Cochrane criteria (Supplementary Figure S7). The domains with the highest proportion of high risk are the performance and detection bias.

## Discussion

The primary findings of this study revealed all six commercially available HIF-PHIs had direct comparisons to ESA and placebo, yet lacked direct comparisons among each other. The reliability of the results from indirect comparisons was confirmed through the consistency test. The network analysis revealed that all six HIF-PHIs effectively increased hemoglobin levels in general CKD patients compared to placebo. However, no significant differences were observed among different HIF-PHIs. These findings were consistent across both the DD and NDD populations. Roxadustat and daprodustat had largest number of reports in terms of adverse events. The overall risk of any AE, SAE, MACE, and mortality did not show an increase when compared to ESA or placebo, and did not vary across different types of HIF-PHIs.

Previous evidence has already demonstrated the effectiveness of HIF-PHIs to treat renal anemia (Holdstock et al., 2016; Zheng et al., 2020; Akizawa et al., 2021a; Yamamoto et al., 2021a; Fadlalmola et al., 2022; Singh et al., 2022; Chen et al., 2023; Yang et al., 2023). Our findings contribute to the existing evidence by providing additional support for the efficacy of these agents and further demonstrating their effectiveness in the general CKD population, as well as across dialysis and non-dialysis dependent populations. Furthermore, a series of systematic reviews (Li et al., 2021; Fatima et al., 2022; Guimarães et al., 2023; Mohamed et al., 2023; Ren et al., 2023; Takkavatakarn et al., 2023; Zheng et al., 2023) have provided compelling evidence for the efficacy of HIF-PHIs. This meta-analysis differs from the literature in several aspects. First, we used network meta-analysis to compare the efficacy and safety of all the commercially available HIF-PHIs so far. Second, this meta-analysis includes a larger number of RCTs and covers the most types of HIF-PHIs in comparison to the published network meta-analysis in this field (Zheng et al., 2020; Fadlalmola et al., 2022; Chen et al., 2023; Yang et al., 2023). Third, compared with this study, the published network meta-analysis either only included DD population (Zheng et al., 2020; Yang et al., 2023), did not include safety outcomes (Chen et al., 2023; Yang et al., 2023), or only analyzed mortality (Zheng et al., 2020). Fourth, we particularly examined the safety outcomes of HIF-PHIs in response to the concerns raised by FDA but did not identify higher MACE risk for HIF-PHIs in CKD population. However, interpretation of these results should be done with caution since the included RCTs were all non-inferiority test in nature. Future studies and long-term surveillance are needed to provide stronger evidence.

The safety of HIF-PHIs has been a subject of considerable scholarly discourse, with specific attention given to the potential cardiovascular risks and elevated levels of VEGF subsequent to treatment (Mima, 2021); yet there exists a dearth of evidence regarding safety outcomes when comparing various types of HIF-PHIs. This study aims to comprehensively assess the occurrences of any AE, cardiovascular AE, severe AE, and mortality following HIF-PHIs treatments. The findings derived from both direct and indirect comparisons in this network analysis provide evidence that the safety profile of currently available HIF-PHIs in the market is not inferior to that of ESA or placebo, in relation to each of the four safety outcomes examined. Although daprodustat and roxadustat had been reported to be associated with increased risk of thrombosis (Chen et al., 2023), this conclusion is limited by the number of studies, thus requiring further substantiation.

The mechanisms underlying the effects of HIF-PHIs in addressing anemia involve the inhibition of prolyl hydroxylase-mediated degradation of HIF-α in response to hypoxia, thereby activating the HIF pathway that regulates erythropoiesis at various levels (Coyne et al., 2011; Koury and Haase, 2015). These mechanisms differ significantly from those of conventional therapies for renal anemia. In addition, the oral administration of HIF-PHIs might help to improve patient compliance and help to reduce the use of medical consumables compared to the subcutaneous administration of ESA in NDD patients.

There are still a few limitations to be mentioned. First, there is a lack of direct comparisons among individual HIF-PHIs, which might have enhanced the evidence should it existed; however, the indirect comparisons are supported by the results of consistency test. Second, it should be noted that although HIF-PHIs has been shown to have advantages in improving iron metabolism in anemia by reducing the hepatic peptide hepcidin (Ganz and Nemeth, 2012), this study did not provide a comprehensive summary of the impact of HIF-PHIs on iron metabolism. Third, epoetin and darbepoetin alpha were considered as a single group in this meta-analysis. Fourth, the evidence of safety outcomes is not long enough due to the limited time after the launch of the first HIF-PHI agent. More stringent evidence is expected with the ongoing post-market surveillance and clinical research.

## Conclusion

In summary, the findings of this systematic review and network meta-analysis suggest that all six commercially available HIF-PHIs effectively increase hemoglobin levels in patients with CKD compared to placebo. No significant differences were observed among the various HIF-PHIs. The overall risk of adverse events, serious adverse events, major adverse cardiovascular events, and mortality did not increase with HIF-PHIs treatment compared to ESA or placebo, and no differences were found among the different types of HIF-PHIs. However, further evidence from long-term studies and the ongoing post-market surveillance is necessary.
