# Supplementary material for: Hypoxia-inducible factor-prolyl hydroxylase inhibitors for treatment of anemia in chronic kidney disease: a systematic review and network meta-analysis
Source: Front Pharmacol. 2024 Jul 10;15:1406588. doi: 10.3389/fphar.2024.1406588 (PMC11267515; doi:10.3389/fphar.2024.1406588)

**Supplementary Materials**

**Supplementary Table 1. Literature search strategy used in this umbrella review.**

Supplementary Table 2. Characteristic s of the included studies.

Supplementary Table 3. Inconsistency test results for all direct and indirect comparisons of efficacy and safety outcomes.

Supplementary Figure 1. Forest plot of direct comparisons for the effects of different treatments on hemoglobin.

Supplementary Figure 2. Comparison for the effect of different treatments on hemoglobin.

and severe adverse event.

Supplementary Figure 3. Forest plot of direct comparisons for any adverse event and severe adverse event.

Supplementary Figure 4. Forest plot of direct comparisons for MACE and mortality.

Supplementary Figure 5. Comparison adjusted funnel plot of the effect on hemoglobin in the included studies.

Supplementary Figure 6. Comparison adjusted funnel plots of the effect on safety outcomes in the included studies.

Supplementary Figure 7. Summary of the risk of bias assessment of the included studies.

**Supplementary Table 1. Literature search strategy used in this umbrella review.**

| Database | Strategies |
| --- | --- |
| Embase via Ovid | 1. HIF-prolyl hydroxylase inhibitor*.mp.  2. Hypoxia-inducible factor.mp.  3. prolyl hydroxylase inhibitor*.mp.  4. HIF-PH inhibitor*.mp.  5. HIF-PHDi.mp.  6. kidney disease.mp.  7. nephropathy.mp.  8. renal disease.mp.  9. anemia/  10. 1 or 2 or 3 or 4 or 5  11. 6 or 7 or 8  12. 9 and 10 and 11  13. random* control* trial.mp.  14. RCT.mp.  15. 13 or 14  16. 12 and 15 |
| Medline via Pubmed | ((HIF-prolyl hydroxylase inhibitor*) OR (Hypoxia-inducible factor) OR (prolyl hydroxylase inhibitor*) OR (HIF-PH inhibitor*) OR (HIF-PHDi)) AND ((kidney disease) OR (nephropathy) OR (renal disease)) AND (anemia) AND ((random* control* trial) OR (RCT)) |
| Cochrane Central Library | ((HIF-prolyl hydroxylase inhibitor*) OR (Hypoxia-inducible factor) OR (prolyl hydroxylase inhibitor*) OR (HIF-PH inhibitor*) OR (HIF-PHDi)) AND ((kidney disease) OR (nephropathy) OR (renal disease)) AND (anemia) AND ((random* control* trial) OR (RCT)) |

**Supplementary Table 2. Characteristic s of the included studies.**

| **Study** | **Nation** | **Registration Number** | **Population** | **Intervention** | | | | **Control** | | | | **Follow-up** | **Outcomes** |
| --- | --- | --- | --- | --- | --- | --- | --- | --- | --- | --- | --- | --- | --- |
|  |  |  |  | Regimen | No. | Age | M/F | Regimen | No. | Age | M/F |  |  |
| Agrawal, 2022 | India | NR | Aged 18-80 years; CKD (Stages 3-5); not on dialysis; Hb level within 7-10g/dl | desidustat 100 mg TIW | 294 | 53.38±13.93 | 147/147 | darbepoetin alfa | 294 | 52.2±13.7 | 149/145 | 24w | HGB/AE/SAE/MACE/mortality |
| Akizawa, 2019 | Japan | NCT01964196 | Aged 20-74 years; not on dialysis; anemia associated with NDD-CKD; Hb levels ≤10 g/dl | roxadustat (50, 70,or 100 mg) TIW | 80 | 64.4±8.7 | 39/41 | placebo | 27 | 61.9±10.6 | 11/16 | 6w | HGB/AE/SAE/mortality |
| Akizawa, 2020 | Japan | NCT02952092 | Aged ≥20 years;HD three times weekly for 12 weeks; Hb level within 10-12g/dl | roxadustat (70 or 100 mg) TIW | 150 | 64.6±11.7 | 101/49 | darbepoetin alfa | 151 | 64.9±10.1 | 107/44 | 24w | HGB/AE/SAE/MACE/mortality |
| Akizawa, 2019 | Japan | NCT02055482 | D 3: patients who either experienced a stopping event in D1 or completed the treatment period in D1 or D2 | molidustat (individually optimized dose) | 118 | 70±10 | 47/61 | darbepoetin | 42 | 69±12 | 22/20 | 36m | HGB |
|  |  | NCT02064426 | D 5: patients who completed the end-of-treatment visit in D4. | molidustat (15, 25, 50, 75, 100, and 150 mg) QD | 57 | 61±12 | 33/24 | epoetin | 30 | 59±9 | 23/7 | 36m | HGB |
| Akizawa, 2019 | Japan | NR | D 1: Anemia associated with CKD; not receiving dialysis | molidustat (25, 50, or 75 mg QD, or 25 or 50 mg BID) | 101 | 68.7±12 | 56/45 | placebo | 20 | 67.1±15.9 | 9/11 | 16w | HGB |
|  |  | NR | D 2: Anemia associated with CKD; previously treated with ESA; not receiving dialysis | molidustat (25, 50, or 75 mg) QD | 92 | 67.6±10.5 | 45/47 | darbepoetin | 32 | 68.8±8.7 | 18/14 | 16w | HGB |
|  |  | NR | D 4: Anemia associated with CKD; previously treated with ESA; receiving regular HD | molidustat(25, 50, 75, or 150 mg) QD | 157 | 59.4±12.5 | 91/66 | epoetin | 42 | 58.9±9.1 | 29/13 | 16w | HGB |
| Akizawa, 2019 | Japan | NR | Aged ≥20 years; HD or HDF 3 times per week for at least 12 weeks; Hb level within 9.5-12g/dl | enarodustat (2, 4, 6 mg) QD | 60 | 62.3±10 | 45/15 | placebo | 22 | 60.7±13 | 15/7 | 30w | HGB/AE/SAE |
| Akizawa, 2021 | Japan | JapicCTI-183938 | Aged ≥20 years; HD 3 times a week at least 12 weeks; Hb level within 9.5-12g/dl | enarodustat 4 mg/d as the initial dose | 86 | 63.2±10.8 | 61/25 | darbepoetin alfa | 86 | 64.8±10.3 | 61/25 | 24w | HGB/AE/SAE/MACE |
| Akizawa, 2021 | Japan | JapicCTI-183870 | Aged ≥20 years; CKD (Stages 3-5); not on dialysis | enarodustat 2 mg/d as the initial dose | 97 | 70.4±9.1 | 61/36 | darbepoetin alfa | 96 | 68.9±9.1 | 47/49 | 24w | HGB/AE/SAE/MACE |
| Akizawa, 2020 | Japan | NCT02969655 | Aged ≥20 years; HD or HDF 3 times per week for at least 12 weeks; Hb level within 9.5-12.5g/dl | daprodustat 4 mg/d as the initial dose | 136 | 64±10 | 91/45 | darbepoetin alfa | 135 | 64±11 | 89/46 | 54w | HGB/AE/SAE |
| Akizawa, 2017 | Japan | NCT02019719 | HD 3 times weekly for ≥ 8 weeks; Hb level within 9.5-12g/dl | daprodustat (4, 6, 8, or 10 mg) QD | 78 | 62.2±9.69 | 38/40 | placebo | 19 | 63.4±8.98 | 12/7 | 4w | HGB/AE/SAE |
| Akizawa, 2012 | Japan | NCT03543657 | Aged ≥20 years; HD or HDF 3 times per week for at least 12 weeks; Hb level within 9.5-12g/dl | molidustat 75 mg/d as the initial dose | 153 | 66.2±10.3 | 91/62 | darbepoetin alfa | 76 | 64.8±10.6 | 49/27 | 52w | HGB/AE/SAE/MACE/mortality |
| Bailey, 2019 | USA | NCT02689206 | Aged ≥18 years; HD 3-5 times weekly for at least 90 days; Hb level within 9.5-11.5g/dl | daprodustat (10, 15, 25 or 30mg) TIW | 79 | 64.1±15 | 50/29 | placebo | 18 | 60.9±13.3 | 7/11 | 24w | HGB/AE/SAE/MACE |
| Barratt, 2021 | UK | NCT02021318 | Aged ≥18 years; CKD (Stages 3-5); not on dialysis; Hb levels ≤10.5 g/dl | roxadustat (70 mg or 100 mg) TIW | 323 | 66.8±13.6 | 145/178 | darbepoetin alfa | 293 | 65.7±14.4 | 129/164 | 104w | AE/SAE/MACE/mortality |
| Barratt, 2021 | UK | NR | Aged ≥18 years; had anemia of CKD, peritoneal dialysis or hemodialysis | roxadustat dose adjustments to maintain Hb between 10-12 g/dl | 2354 | 55.5±14.9 | 1365/989 | ESA | 2360 | 56.3±14.6 | 1379/981 | 36w | HGB/MACE/mortality |
| Besarab, 2015 | USA | NCT00761657 | Aged 18−80 years; CKD (Stages 3-4); not on dialysis | roxadustat(1, 1.5, 2 and 0.7 mg/kg) BIW or TIW | 88 | 64 | 33/55 | placebo | 28 | 68.6 | 16/12 | 12w | AE/SAE |
| Brigandi, 2016 | USA | NCT01047397 | Aged 18−85 years; CKD (Stages 3-5); not on dialysis; Hb levels≤11.0 g/dl | daprodustat (10, 25, 50 or 100mg) QD | 61 | 59.4±13.5 | 27/34 | placebo | 9 | 54.7±17.3 | 6/3 | 2m | MACE |
| Charytan, 2021 | USA | NCT02273726 | Aged ≥18 years; dialysis for ≥3months before screening; Hb level within 9-12g/dl | roxadustat (70, 100, 150 or 200mg) TIW | 370 | 57.6±13.6 | 187/183 | epoetin alfa | 371 | 58.4±13.3 | 215/156 | 52w | HGB/AE/MACE |
| Chen, 2019 | China | NCT02652806 | Aged 18−75 years; dialysis for at least 16 weeks; Hb level within 9-12g/dl | roxadustat 100mg or 120 mg as the starting dose | 204 | 47.6±11.7 | 126/78 | epoetin alfa | 100 | 51±11.8 | 58/42 | 27w | HGB/AE/SAE/MACE |
| Chen, 2019 | China | NCT02652819 | Aged 18−75 years; CKD (Stages 3-5); not on dialysis; Hb level within 7-10g/dl | roxadustat (70 or 100mg) TIW | 101 | 54.7±13.3 | 36/65 | placebo | 51 | 53.2±13.1 | 20/31 | 9w | HGB/AE/SAE/MACE |
| Chen, 2017 | China | NCT01599507 | Aged 18−80 years; CKD (Stages 3-5); not on dialysis; Hb levels≤10 g/dl | roxadustat low-dose(1.1-1.75mg/kg) or high-dose (1.5-2.25mg/kg) TIW | 61 | 48.8±13.8 | 18/43 | placebo | 30 | 51.4±11.9 | 8/22 | 8w | HGB/AE/SAE |
|  |  | NCT01596855 | Aged 18−80 years; received dialysis; Hb level within 9-12g/dl | roxadustat low-dose(1.1-1.8mg/kg) or meduim-dose (1.5-2.3mg/kg) or high-dose (1.7-2.3mg/kg) TIW | 74 | 49.9±12.8 | 45/29 | epoetin alfa | 22 | 53.8±10 | 13/9 | 6w | HGB/AE/SAE |
| Chertow, 2021 | USA | NCT02648347 | Aged ≥18 years; not on dialysis; Hb levels≤10 g/dL; had not received previous treatment with ESAs | vadadustat 300mg as the starting dose | 879 | 65.2±14.3 | 404/475 | darbepoetin alfa | 872 | 64.9±13.7 | 366/506 | 58m | HGB/AE/SAE/mortality |
|  |  | NCT02680574 | Aged ≥18 years; not on dialysis; Hb levels≤10 g/dL; had treated with ESAs | vadadustat 300mg as the starting dose | 862 | 67.3±13.1 | 394/468 | darbepoetin alfa | 863 | 66.5±13.5 | 375/488 | 58m | HGB/AE/SAE/mortality |
| Coyne, 2021 | USA | NCT01750190 | Aged ≥18 years; CKD (Stages 3-5); not on dialysis; Hb levels≤10g/dl | roxadustat (70 or 100mg) TIW | 616 | 64.9±12.6 | 241/375 | placebo | 306 | 64.8±13.2 | 130/176 | 240w | AE/SAE/MACE |
| Coyne, 2022 | USA | NCT03400033 | HD 3 times per week for at least 90 days; Hb level within 8-11.5g/dl | daprodustat 2-48mg TIW | 270 | 60 | 149/121 | epoetin | 137 | 56 | 81/56 | 52w | HGB/AE/SAE/MACE/mortality |
| Csiky, 2021 | Hungary | NCT02964936 | Aged ≥18 years; HD/PD for at least 4 months; Hb level within 9.5-12g/dl | roxadustat (20,50,100mg) TIW | 414 | 61±13.8 | 245/169 | ESA | 420 | 61.8±13.4 | 235/185 | 52w | HGB/AE/SAE/mortality |
| Eckardt, 2021 | Germany | NCT02865850 | Aged ≥18 years; undergoing dialysis;Hb level within 8-11g/dl | vadadustat 300mg as the starting dose | 181 | 56.5±14.8 | 107/74 | darbepoetin alfa | 188 | 55.6±14.6 | 113/75 | 44m | HGB/AE/SAE/MACE/mortality |
|  |  | NCT02892149 | Aged ≥18 years; undergoing dialysis;Hb level within 8-11g/dl | vadadustat 300mg as the starting dose | 1777 | 57.9±13.9 | 990/787 | darbepoetin alfa | 1777 | 58.4±13.8 | 1004/773 | 44m | HGB/AE/SAE/MACE/mortality |
| Fishbane, 2021 | USA | NCT02174627 | Aged ≥18 years; CKD (Stages 3-5); not on dialysis; Hb level < 10g/dl | roxadustat 70mg TIW as the starting dose | 1384 | 60.9±14.7 | 564/820 | placebo | 1377 | 62.4±14.1 | 603/774 | 36m | HGB/AE/SAE/MACE/mortality |
| Fishbane, 2022 | USA | NCT02174731 | Aged ≥18 years;HD or PD for ≥30 days before randomization; Hb level within 10-12g/dl | Roxadustat 70-200mg TIW as the starting dose | 1051 | 53.5±15.3 | 625/426 | epoetin alfa | 1055 | 54.5±15 | 626/429 | 164w | HGB/AE/SAE/MACE/mortality |
| Gang, 2022 | India | CTRI/2019/12/022312 | Aged ≥18 years;on dialysis (≥2 times in a week) for at least 12 weeks; baseline Hb level within 8-11g/dl | desidustat 100 mg TIW | 196 | 51.02±13.97 | 135/61 | epoetin alfa | 196 | 50.91±13.48 | 134/62 | 24w | HGB/AE/SAE/mortality |
| Holdstock, 2019 | USA | NCT01977573 | CKD (Stages 3-5); not on dialysis | daprodustat (1, 2 or 4 mg) QD | 156 | 66.5±12.78 | 64/92 | rhEPO | 79 | 65.4±13.6 | 33/46 | 24w | AE/SAE/MACE/mortality |
| Holdstock, 2016 | USA | NCT01587898 | Aged ≥18 years; CKD (Stages 3-5); not on dialysis and were not taking rhEPO; Hb level within 8.5-11g/dl | daprodustat (0.5, 2, 5mg) QD | 54 | 68.3±11.5 | 16/38 | placebo | 18 | 69.2±11 | 4/14 | 4w | HGB/SAE |
|  |  | NCT01587924 | Aged ≥18 years; HD 3 times weekly for at least 8 weeks, and were using rhEPO;Hb level within 9.5-12.0 g/dl | daprodustat (0.5, 2, 5mg) QD | 62 | 55.7±17.7 | 43/19 | rhEPO | 20 | 64.2±12.8 | 16/4 | 4W | HGB/SAE |
| Hou, 2022 | China | ChiCTR2000035054 | Patients diagnosed with CKD and renal anemia; received PD; Hb levels ≤12 g/dL | roxadustat (100 or 120mg) TIW | 86 | 48±12 | 47/39 | ESAs | 43 | 48.3±13 | 25/18 | 24w | HGB/AE/SAE/MACE |
| Martin, 2017 | USA | NCT01381094 | Aged 18−79 years; CKD (Stages 3-4); Hb levels≤10.5 g/dl | vadadustat (240, 370, 500, or 630 mg) QD | 72 | 65.7±9.7 | 36/36 | placebo | 19 | 64.9±10 | 7/12 | 6w | HGB/AE/SAE/mortality |
| Meadowcroft, 2019 | USA | NCT01977482 | HD 3-5 times weekly; Hb level within 9-11.5g/dl | daprodustat (4, 6, 8, 10 or 12mg) QD | 171 | 59.6±13.3 | 108/63 | placebo | 39 | 59.7±18.7 | 26/13 | 24w | HGB/AE/MACE/mortality |
| Nangaku, 2020 | Japan | NCT03054337 | Aged ≥20 years; not on dialysis; BMI≤42.0 kg/m2 | vadadustat (150, 300 or 600 mg) QD | 37 | 69.8±11.7 | 19/18 | placebo | 14 | 71.4±11.6 | 10/4 | 16w | HGB/AE |
|  |  | NCT03054350 | Aged ≥20 years; on dialysis; BMI≤42.0 kg/m2 | vadadustat (150, 300 or 600 mg) QD | 44 | 63.3±9.2 | 32/12 | placebo | 14 | 65.7±11.6 | 8/6 | 16w | HGB/AE |
| Nangaku, 2021 | Japan | NCT02791763 | CKD (Stages 3-5); not on dialysis; Hb level within 8-11g/dl(ESA-naïve) or 9-13g/dl(ESA users) | daprodustat 2-4 mg, QD | 108 | 69±11 | 66/42 | ESA | 109 | 71±9 | 69/40 | 52w | HGB/AE/MACE |
| Nangaku, 2021 | Japan | NCT03329196 | Aged ≥20 years; on dialysis; Hb level within 8-11g/dl(ESA-naïve) or 9-12.5g/dl(ESA users) | vadadustat 300mg qd as the starting dose | 151 | 71.7±10.3 | 75/76 | darbepoetin alfa | 153 | 72.2±9.5 | 73/80 | 52w | HGB/AE/SAE/MACE/mortality |
| Nangaku | Japan | NCT03439137 | Aged ≥20 years; HD or HDF 3 times a week for 12 weeks; Hb level within 9.5-12g/dl | vadadustat was started at 300 mg once daily, and the dose was adjusted to 150–600 mg | 162 | 66±11.3 | 104/58 | darbepoetin alfa | 161 | 64.9±11.7 | 109/52 | 52w | HGB/AE/SAE/MACE/mortality |
| Parmar, 2019 | India | CTRI/2017/05/008534 | Aged 18-65 years; CKD (Stages 1-4); Hb level within 6.5-11g/dl | desidustat (100, 150, or 200 mg) TIW | 87 | 48.48±12.1 | 33/54 | placebo | 30 | 46.9±12.66 | 17/13 | 6w | HGB/AE |
| Pergola, 2016 | USA | NCT01906489 | Aged ≥20 years; CKD (Stages 3-5); not on dialysis | vadadustat 150-600mg QD | 138 | 66.6±9.97 | 57/81 | placebo | 72 | 65.9±12.33 | 38/34 | 16w | AE/SAE/mortality |
| Provenzano, 2016 | USA | NCT01147666 | Aged 18-75 years; HD 3 times a week for 4 or more months. Hb levels were 9.0-13.5 g/dl | roxadustat (1.0, 1.5, 1.8, or 2.0 mg/kg) TIW | 67 | 56.9±12.1 | 45/22 | epoetin alfa | 23 | 57±11.6 | 14/9 | 19w | HGB/AE/SAE/MACE |
| Provenzano, 2021 | USA | NCT02052310 | Aged ≥18 years; HD or PD for 2 weeks to ≤ 4 months; Hb levels ≤10g/dl. | roxadusta (70 or 100mg) TIW | 522 | 53.8±14.7 | 309/213 | epoetin alfa | 521 | 54.3±14.6 | 307/214 | 52w | HGB/SAE/MACE/mortality |
| Shutov, 2021 | Russia | NCT01887600 | Aged ≥18 years; diagnosed with CKD stage 3-5; not on dialysis;Hb levels ≤10g/dl. | roxadusta (70 or 100mg) TIW | 391 | 62 | 169/222 | placebo | 203 | 63 | 99/104 | 104w | HGB/AE/SAE/MACE/mortality |
| Singh, 2021 | USA | NCT02876835 | CKD (Stages 3-5); not on dialysis | daprodustat 1-4mg QD | 1937 | 67 | 835/1102 | darbepoetin alfa | 1935 | 67 | 864/1071 | 148w | HGB/AE/SAE/MACE/mortality |
| Singh, 2021 | USA | NCT02879305 | Dialysis for at least 90 days; Hb levels were 8-11.5g/dl | daprodustat 4-12mg QD | 1487 | 58 | 851/636 | ESA | 1477 | 59 | 847/630 | 148w | HGB/AE/SAE/MACE/mortality |
| Singh, 2022 | USA | NCT03029208 | Started and received HD or PD within 90 days before randomization; Hb levels were 8-10.5g/dl | daprodustat 2mg QD | 157 | 52 | 96/61 | darbepoetin alfa | 155 | 56 | 98/57 | 52w | HGB/AE/SAE/MACE/mortality |
| Yamamoto, 2021 | Japan | NCT03350347 | Aged ≥20 years; CKD (Stages 3-5); not on dialysis;Hb levels were 10-13g/dl | molidustat 25 or 75mg QD at a starting dose | 82 | 69±10.3 | 45/37 | darbepoetin | 82 | 72.4±10.3 | 54/28 | 32w | HGB/AE/SAE/MACE/mortality |
| Yamamoto, 2021 | Japan | NCT03350321 | Aged ≥20 years; CKD (Stages 3-5); not on dialysis;Hb levels were 8-11g/dl | molidustat 25 QD at a starting dose | 82 | 72.1±9.3 | 50/32 | darbepoetin | 80 | 71.2±10.1 | 50/30 | 52w | HGB/AE/SAE/mortality |

**Supplementary Table 3. Inconsistency test results for all direct and indirect comparisons of efficacy and safety outcomes.**

| Treatments | Direct Coef. | Std. Err. | Indirect Coef. | Std. Err. | Difference Coef. | Std. Err. | P>\|z\| | Tau |
| --- | --- | --- | --- | --- | --- | --- | --- | --- |
| Hemoglobin | | | | | | | | |
| Placebo vs Roxadustat | 1.402 | 0.111 | 1.375 | 0.123 | 0.027 | 0.166 | 0.871 | 0.211 |
| Placebo vs Molidustat | 1.200 | 0.359 | 1.220 | 0.125 | -0.020 | 0.380 | 0.958 | 0.209 |
| Placebo vs Vadadustat | 1.389 | 0.139 | 1.005 | 0.121 | 0.384 | 0.184 | 0.037 | 0.197 |
| Placebo vs Desidustat | 1.780 | 0.428 | 1.273 | 0.176 | 0.507 | 0.462 | 0.273 | 0.207 |
| Placebo vs Daprodustat | 1.036 | 0.136 | 1.236 | 0.122 | -0.199 | 0.183 | 0.275 | 0.201 |
| Placebo vs Enarodustat | 1.510 | 0.320 | 1.435 | 0.189 | 0.075 | 0.372 | 0.840 | 0.209 |
| ESA vs Roxadustat | 0.222 | 0.068 | 0.237 | 0.152 | -0.015 | 0.166 | 0.927 | 0.211 |
| ESA vs Molidustat | 0.054 | 0.095 | 0.033 | 0.368 | 0.021 | 0.380 | 0.956 | 0.209 |
| ESA vs Vadadustat | -0.071 | 0.084 | 0.278 | 0.164 | -0.349 | 0.184 | 0.058 | 0.197 |
| ESA vs Desidustat | 0.124 | 0.156 | 0.613 | 0.434 | -0.489 | 0.461 | 0.290 | 0.207 |
| ESA vs Daprodustat | 0.042 | 0.083 | -0.253 | 0.162 | 0.295 | 0.182 | 0.104 | 0.199 |
| ESA vs Enarodustat | 0.273 | 0.171 | 0.348 | 0.331 | -0.075 | 0.372 | 0.840 | 0.209 |
| Any AE | | | | | | | | |
| Placebo vs Roxadustat | 0.032 | 0.013 | -0.032 | 0.058 | 0.064 | 0.059 | 0.280 | 0.009 |
| Placebo vs Vadadustat | 0.017 | 0.078 | 0.009 | 0.019 | 0.008 | 0.080 | 0.918 | 0.010 |
| Placebo vs Desidustat | -0.372 | 0.453 | 0.001 | 0.068 | -0.372 | 0.458 | 0.416 | 0.009 |
| Placebo vs Daprodustat | -0.071 | 0.091 | 0.043 | 0.021 | -0.114 | 0.094 | 0.226 | 0.008 |
| Placebo vs Enarodustat | -0.257 | 0.206 | -0.039 | 0.053 | -0.218 | 0.213 | 0.305 | 0.009 |
| ESA vs Roxadustat | 0.009 | 0.012 | 0.073 | 0.058 | -0.064 | 0.059 | 0.280 | 0.009 |
| ESA vs Molidustat | -0.005 | 0.020 | -0.032 | 7.557 | 0.027 | 7.557 | 0.997 | 0.009 |
| ESA vs Vadadustat | -0.009 | 0.009 | -0.001 | 0.079 | -0.008 | 0.080 | 0.918 | 0.010 |
| ESA vs Desidustat | -0.018 | 0.066 | -0.390 | 0.453 | 0.372 | 0.458 | 0.416 | 0.009 |
| ESA vs Daprodustat | 0.021 | 0.014 | -0.092 | 0.093 | 0.114 | 0.094 | 0.226 | 0.008 |
| ESA vs Enarodustat | -0.058 | 0.050 | -0.276 | 0.207 | 0.218 | 0.213 | 0.305 | 0.009 |
| SAE | | | | | | | | |
| Placebo vs Roxadustat | 0.071 | 0.047 | 0.486 | 0.273 | -0.415 | 0.277 | 0.134 | 0.053 |
| Placebo vs Vadadustat | 0.462 | 0.306 | -0.012 | 0.069 | 0.475 | 0.314 | 0.131 | 0.052 |
| Placebo vs Daprodustat | -0.085 | 0.601 | 0.093 | 0.073 | -0.178 | 0.606 | 0.769 | 0.052 |
| Placebo vs Enarodustat | 2.517 | 1.458 | 0.104 | 0.262 | 2.413 | 1.481 | 0.103 | 0.052 |
| ESA vs Roxadustat | 0.061 | 0.040 | -0.354 | 0.274 | 0.415 | 0.277 | 0.134 | 0.053 |
| ESA vs Molidustat | 0.098 | 0.105 | -0.071 | 36.264 | 0.169 | 36.265 | 0.996 | 0.052 |
| ESA vs Vadadustat | -0.025 | 0.034 | 0.450 | 0.312 | -0.475 | 0.314 | 0.131 | 0.052 |
| ESA vs Desidustat | 0.247 | 0.262 | -0.073 | 185.446 | 0.320 | 185.446 | 0.999 | 0.052 |
| ESA vs Daprodustat | 0.062 | 0.045 | -0.116 | 0.604 | 0.178 | 0.606 | 0.769 | 0.052 |
| ESA vs Enarodustat | 0.079 | 0.255 | 2.491 | 1.459 | -2.412 | 1.481 | 0.103 | 0.052 |
| MACE | | | | | | | | |
| Placebo vs Roxadustat | 0.067 | 0.181 | 0.218 | 0.881 | -0.151 | 0.900 | 0.867 | 0.000 |
| Placebo vs Daprodustat | 0.280 | 0.879 | 0.129 | 0.196 | 0.151 | 0.900 | 0.866 | 0.000 |
| ESA vs Roxadustat | -0.078 | 0.061 | -0.229 | 0.898 | 0.151 | 0.900 | 0.867 | 0.000 |
| ESA vs Molidustat | 0.217 | 0.825 | -0.388 | 356.232 | 0.605 | 356.233 | 0.999 | 0.000 |
| ESA vs Vadadustat | -0.050 | 0.065 | -0.292 | 104.757 | 0.243 | 104.757 | 0.998 | 0.000 |
| ESA vs Desidustat | -1.386 | 1.115 | -0.751 | 682.186 | -0.635 | 682.186 | 0.999 | 0.000 |
| ESA vs Daprodustat | -0.017 | 0.044 | 0.134 | 0.899 | -0.151 | 0.900 | 0.867 | 0.000 |
| Mortality | | | | | | | | |
| Placebo vs Roxadustat | 0.127 | 0.135 | 0.595 | 0.888 | -0.468 | 0.898 | 0.602 | 0.112 |
| Placebo vs Vadadustat | 0.605 | 1.103 | 0.295 | 0.178 | 0.310 | 1.118 | 0.782 | 0.112 |
| Placebo vs Daprodustat | 0.905 | 1.471 | 0.174 | 0.180 | 0.731 | 1.482 | 0.622 | 0.111 |
| ESA vs Roxadustat | -0.010 | 0.079 | -0.478 | 0.895 | 0.468 | 0.898 | 0.602 | 0.112 |
| ESA vs Molidustat | 0.358 | 0.677 | -0.160 | 255.555 | 0.519 | 255.557 | 0.998 | 0.111 |
| ESA vs Vadadustat | 0.150 | 0.084 | 0.459 | 1.114 | -0.310 | 1.118 | 0.782 | 0.112 |
| ESA vs Desidustat | -0.258 | 0.427 | -0.300 | 283.484 | 0.041 | 283.484 | 1.000 | 0.111 |
| ESA vs Daprodustat | 0.031 | 0.095 | 0.761 | 1.479 | -0.731 | 1.482 | 0.622 | 0.111 |

Note: P > 0.05 indicates consistency among the direct and indirect comparison, while p<0.05 indicated high inconsistency among the direct and indirect comparison.

**Supplementary Figure 1. Forest plot of direct comparisons for the effects of different treatments on hemoglobin.**


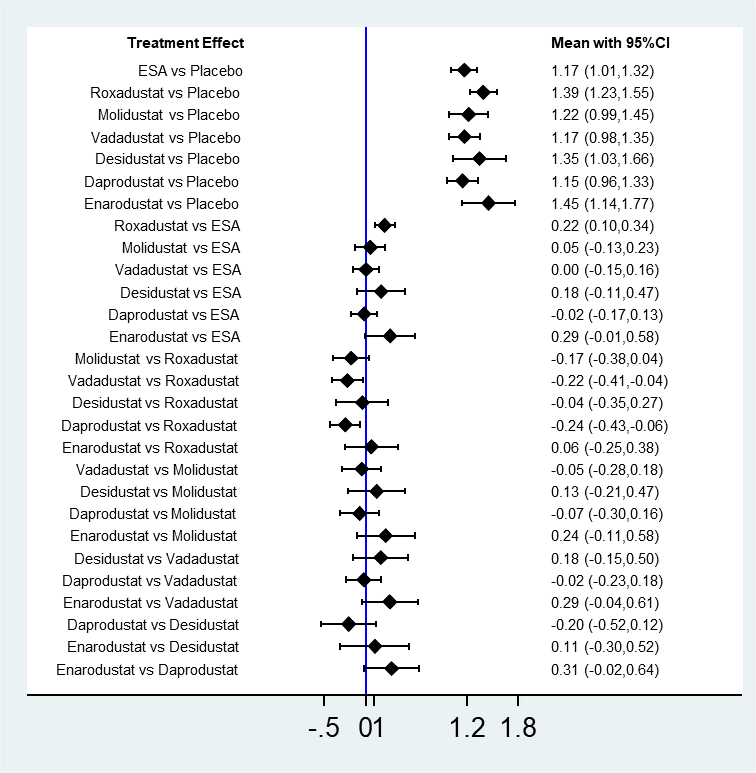


Abbreviations: ESA, erythropoietin stimulation agents.

**Supplementary Figure 2. Comparison for the effect of different treatments on hemoglobin.**


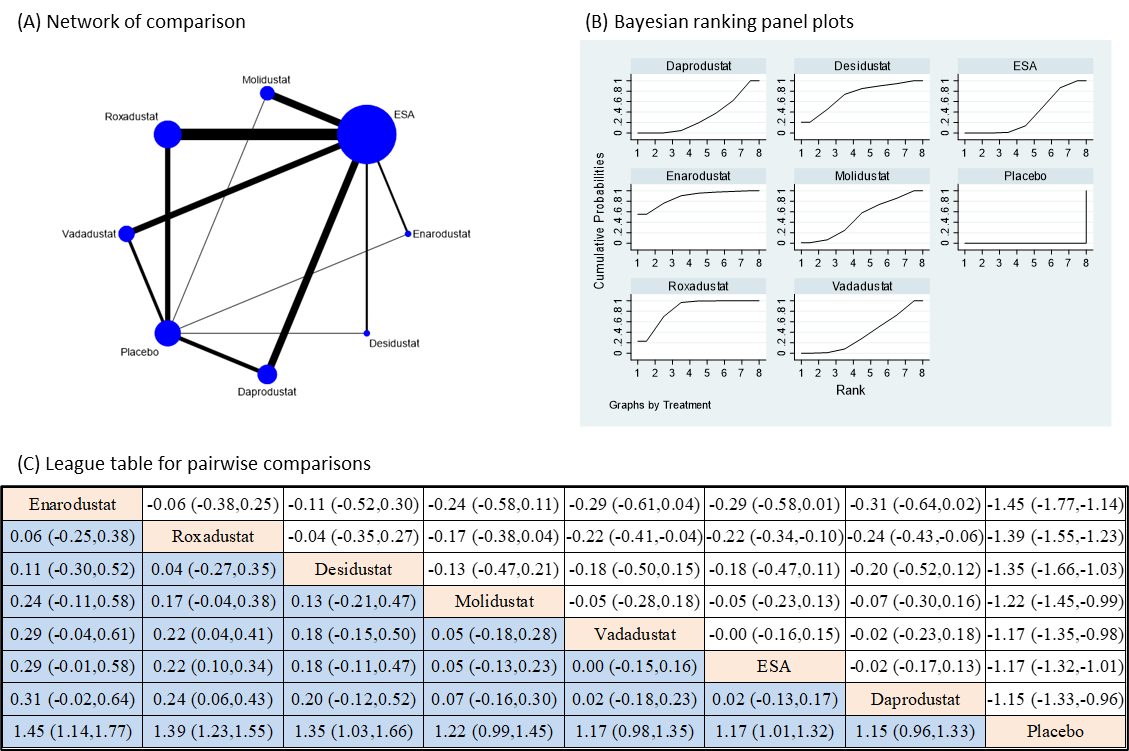


Note: (A) In the network of comparisons, the size of nodes is proportional to the total sample size of each treatment, and the width of lines is proportional to the number of studies in each pair of comparison. (B) Bayesian ranking panel plots indicate the higher the rank reflected by the area under curve, the superior the treatment to increase the levels of hemoglobin. (C) The league table of pairwise comparison for the effects of different treatments on hemoglobin levels indicated the greatest relative increase in hemoglobin was observed following enarodustat compared to placebo. All treatments are ordered based on efficacy ranking.

**Supplementary Figure 3. Forest plot of direct comparisons for any adverse event** **and severe adverse event.**


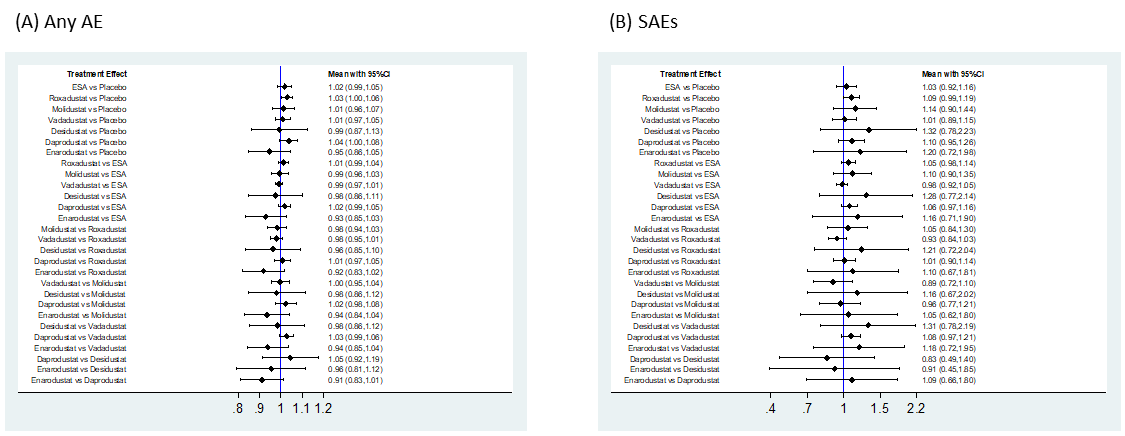


Abbreviations: ESA, erythropoietin stimulation agents.

**Supplementary Figure 4. Forest plot of direct comparisons for MACE and mortality.**


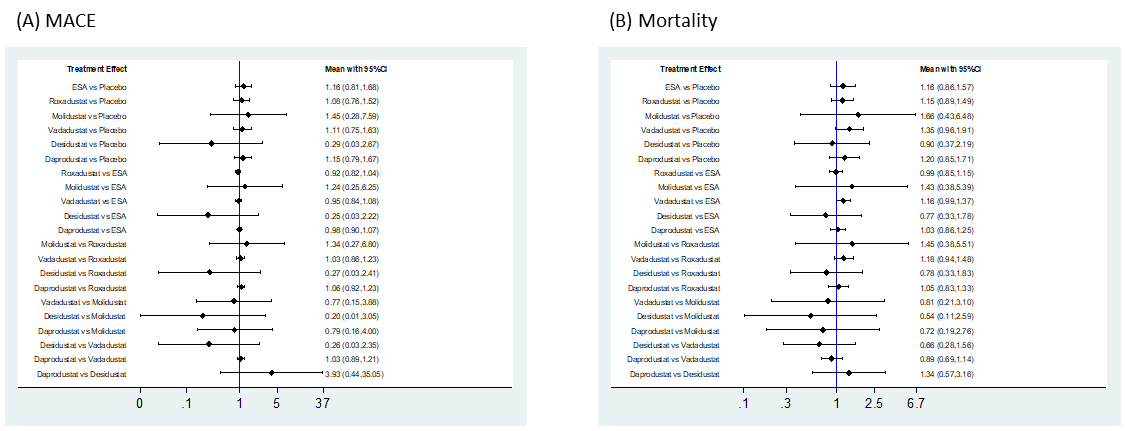


Abbreviations: ESA, erythropoietin stimulation agents.

**Supplementary Figure 5. Comparison adjusted funnel plot of the effect on hemoglobin in the included studies.**


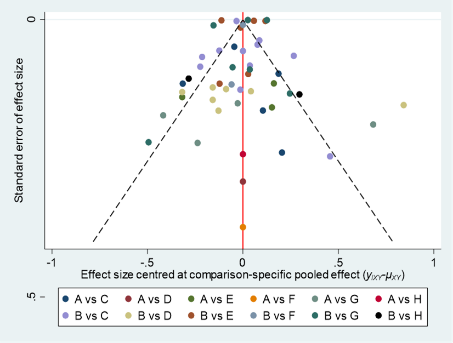


Note: The treatments are labeled as follow:1) A: placebo; 2) B: ESA; 3) C: roxadustat; 4) D: molidustat; 5) E: vadadustat; 6) F: desidustat; 7) G: daprodustat; 8) H: enarodustat.

Abbreviations: ESA, erythropoietin stimulation agents.

**Supplementary Figure 6. Comparison adjusted funnel plots of the effect on safety outcomes in the included studies.**


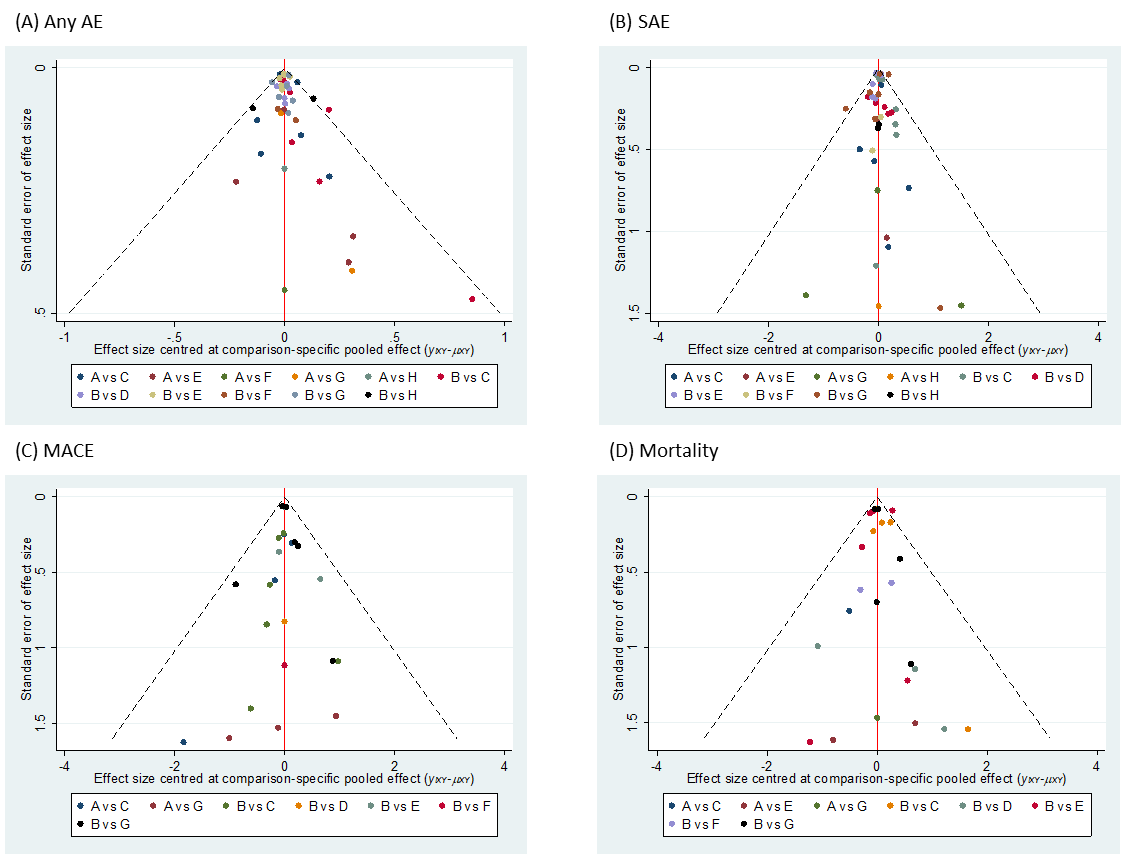


Note: The treatments are labeled as follow:1) A: placebo; 2) B: ESA; 3) C: roxadustat; 4) D: molidustat; 5) E: vadadustat; 6) F: desidustat; 7) G: daprodustat; 8) H: enarodustat.

Abbreviations: AE, adverse event; ESA, erythropoietin stimulation agents; MACE, major adverse cardiovascular event; SAE, severe adverse event.

**Supplementary Figure 7. Summary of the risk of bias assessment of the included studies.**


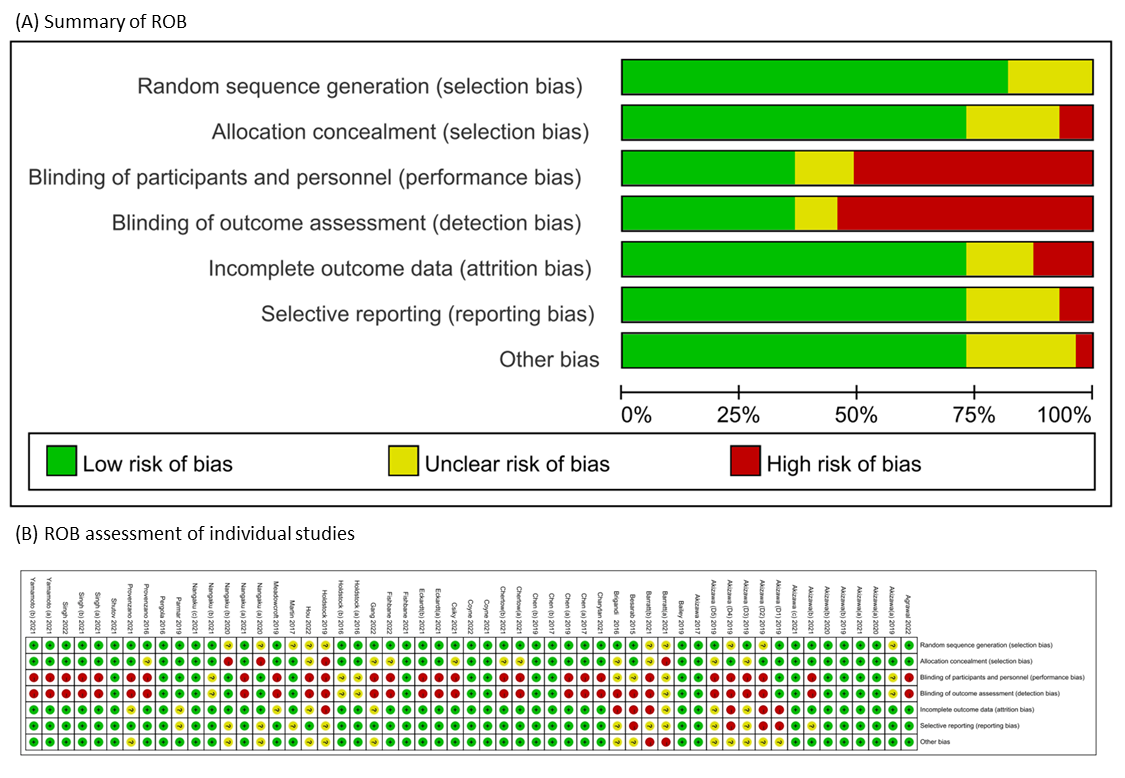

Supplement: Supplementary file 1 [file DataSheet1.docx]
